# Outcome and complications in infants with respiratory failure: venovenous two-site versus double-lumen ECMO

**DOI:** 10.1186/cc9590

**Published:** 2011-03-11

**Authors:** M Hermon, G Mostafa, J Golej, G Burda, R Vargha, G Trittenwein

**Affiliations:** 1 Medical University of Vienna, Austria

## Introduction

Extracorporeal membrane oxygenation (ECMO) provides temporary life support for children with severe respiratory or cardiac failure. Since 1990, more than 27,000 children have received ECMO and an overall survival rate of 76% [1] has been observed. The objective of this study was to compare outcomes and complications of the two-site venovenous versus the double-lumen ECMO in infants with respiratory failure.

## Methods

The Extracorporeal Life Support Organization (ELSO, Ann Arbor, MI, USA) registry database collected between 1999 and 2009 was provided for research. A total of 9,086 children ≤7 kg BW were treated with ECMO. From these children, those who were older than 32 days and received VV ECMO were extracted for analysis. A total of 270 children met the inclusion criteria. Two hundred and thirty-six children were treated with VVDL ECMO and 34 children received VV two-site ECMO. ELSO registry records were reviewed for the following information: demographic data, type of ventilation, ventilator days and settings during an ECMO run, complications during an ECMO run and outcome.

## Results

In this study 87% (*n *= 236) of the children were cannulated with VVDL and 13% (*n *= 34) using the VV two-site technique. APGAR scores were significantly lower in the VV two-site group. Twenty-four hours after ECMO onset, ventilator settings were significantly higher in the VV two-site group. ECMO duration was significantly shorter in the VV two-site group (137 hours vs. 203 hours, *P *< 0.01). The total complication rate, however, did not differ between the groups. Survival rates (71% in the VVDL group and 56% in the VV group) were not significantly different either.

## Conclusions

The total complication rate was found to be similar in both groups. The ECMO duration period was significantly shorter in the VV two-site group. No difference was found in survival rates between the two groups. Neither of the two-cannulation methods - venovenous two-site or venovenous double-lumen ECMO - has shown any significant superiority. The decision about which technique to use for infants depends mainly on the best practice experience of each individual ECMO centre and their routinely-used technical equipment.
